# Centromedian thalamic nucleus with or without anterior thalamic nucleus deep brain stimulation for epilepsy in children and adults: A retrospective case series

**DOI:** 10.1016/j.seizure.2020.11.012

**Published:** 2020-11-26

**Authors:** Juan Luis Alcala-Zermeno, Nicholas M. Gregg, Elaine C. Wirrell, Matt Stead, Gregory A. Worrell, Jamie J. Van Gompel, Brian Nils Lundstrom

**Affiliations:** a Department of Neurology, Mayo Clinic, Rochester, MN, USA; b Division of Child and Adolescent Neurology, Department of Neurology, Mayo Clinic, Rochester, MN, USA; c Dark Horse Neuro, Inc, Bozeman, MT, USA; d Department of Neurologic Surgery, Mayo Clinic, Rochester, MN, USA

**Keywords:** Centromedian, Anterior nucleus, Deep brain stimulation, Epilepsy, Seizure, Thalamus

## Abstract

The centromedian (CM) and anterior nucleus of the thalamus (ANT) are deep brain stimulation (DBS) targets for management of generalized, and focal drug resistant epilepsy (DRE), respectively. We report on a single center retrospective case series of 16 children and adults with DRE who underwent CM with simultaneous ANT (69 %) or CM without simultaneous ANT DBS (31 %). Seizure frequency, epilepsy severity, life satisfaction, and quality of sleep before and after DBS were compared. Baseline median seizure frequency was 323 seizures per month (IQR, 71–563 sz/mo). Median follow up time was 80 months (IQR 37–97 mo). Median seizure frequency reduction was 58 % (IQR 13–87 %, p = 0.002). Ten patients (63 %) reported ≥50 % seizure frequency reduction. Median seizure frequency reduction and responder rate were not significantly different for CM + ANT versus CM only. Seizure severity and life satisfaction were significantly improved. Three patients (19 %) developed device-related side effects, 2 of them (12.5 %) required surgical intervention. In a heterogenous population of children and adults with generalized, multifocal, posterior onset, and poorly localized DRE, CM with or without ANT DBS is feasible, relatively safe and is associated with reduced seizure frequency and severity, as well as improved life satisfaction.

## 1. Introduction

There are over 45.9 million people living with epilepsy worldwide [1]. Around 30 % of patients with epilepsy are drug-resistant [2]. Decreased quality of life, medication side effects, psychiatric comorbidities, and increased health-care utilization are burdens of living with epilepsy [3,4]. Individuals with drug-resistant epilepsy (DRE) are at higher risk of epilepsy-related morbidity and mortality [5,6]. Epilepsy surgery remains the cornerstone of DRE management, but treatment options are limited for patients whose seizures have difficult to localize onset zones, when epilepsy surgery has failed to control seizures, or when they have multifocal or generalized onset seizures.

There are several neuromodulation strategies available for epilepsy management including vagus nerve stimulation (VNS), responsive neurostimulation (RNS), and deep brain stimulation (DBS). Stimulation of anterior nucleus of thalamus for epilepsy (SANTE) trial demonstrated the efficacy of anterior thalamic nuclei stimulation (ANT) as a treatment modality for frontal or temporal DRE and is FDA approved for treatment of focal seizures [7,8]. In generalized epilepsy, centromedian thalamic DBS (CM) may disrupt the thalamocortical feedback loops responsible for generalized hypersynchrony [9]. CM DBS has shown efficacy in patients with generalized epilepsy, although it is not an FDA-approved approach; CM DBS has been less effective in patients with frontal or temporal lobe DRE in small controlled clinical trials [10,11].

Patients with multifocal, posterior onset, or poorly localized epileptogenic foci are challenging to treat, and therapeutic options for these patients are limited [8,10,11]. Moreover, patients with generalized DRE are a heterogeneous population that may have variable responses to deep brain stimulation [12]. The aim of this study is to report the clinical efficacy of simultaneous CM + ANT stimulation as well as exclusive CM stimulation for patients with DRE managed in a large tertiary care center.

## 2. Methods

### 2.1. Study design

Retrospective review and standardized questionnaire assessment of consecutive patients (n = 16) that were implanted with DBS targeting CM with (n = 11) or without (n = 5) simultaneous ANT implantation at our institution from 2010–2018, for the management of DRE.

### 2.2. Patients

**Fig. 1.**
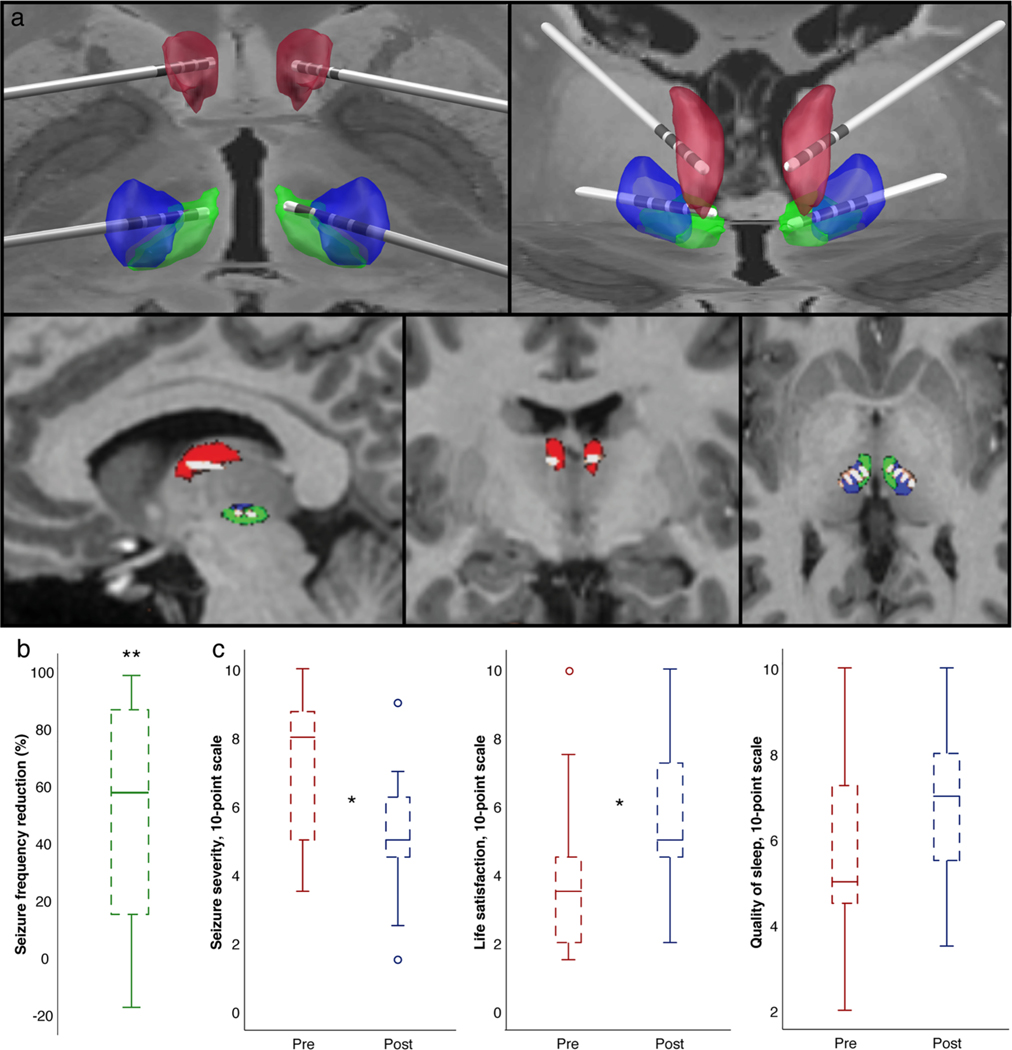
Example trajectories from patient 11 (posterior transcortical approach) and patient outcomes. (a) Lead trajectories for a single patient. Red represents ANT, Blue represents CM and green represents parafascicular nucleus. Views in order of appearance: posterior 3D view, anterior 3D view, Sagittal 2D, Coronal 2D, Axial 2D. (b) Seizure frequency reduction (n = 16). (c) Patient reported outcomes before and after DBS for seizure severity (1 = mildest), life satisfaction (10 = best), and quality of sleep (10 = best). * signifies p < 0.05 and ** p < 0.01.

DRE was defined by ILAE criteria [13]. Before DBS implantation, patients were evaluated with prolonged video EEG and epilepsy protocol MRI. All patients were discussed in a multidisciplinary epilepsy surgery and neuromodulation committee. Patients with generalized and/or multifocal epilepsy were candidates for CM implantation. The addition of ANT stimulation was proposed with the goal of maximizing seizure reduction based on expert opinion from the neuromodulation committee. Presurgical stereotactic MRI images were performed after Leksell frame and MRI localizer box (Elekta Instruments, Atlanta, GA) fixation. For ANT targeting, typical approaches include either a transventricular or frontal extraventricular approach. We used the latter approach for 10 of 11 CM + ANT (4 leads) patients according to Medtronic recommendations and adjusting via the Schaltenbrand and Wahren atlas [14]. In recent years, we have employed an alternate posterior parietal extraventricular approach, which appears to be safe and efficacious [15]. With this approach the electrode is placed in an approximate axial plane with the distal contacts targeting a location 2 mm superior to the mammillothalamic tract, as assessed by MR T1 inversion recovery (FGATIR) imaging. This approach was used for one CM + ANT patient (Fig. 1). CM was targeted similar to prior studies [16]: The nucleus was targeted as 5−12 mm lateral from midline, 0−2 mm superior to the anterior commissure-posterior commissure (AC-PC) line, and either 8 mm posterior to the AC-PC midpoint or just anterior to PC. Electrode trajectories were 45–60 degrees from the AC-PC line. Off-label FDA-approved Medtronic DBS stimulation hardware (Medtronic, Minneapolis, MN, USA) was utilized. In 7/11 CM + ANT cases only one device with capability of four lead stimulation was implanted, while in 4/11 CM + ANT patients two stimulation devices were implanted. All CM only cases (5/5) were each implanted with a single stimulation device (Table 1). Stimulation parameter capabilities are similar for all devices used. Postoperative MRI or CT were used for target confirmation. DBS settings were programmed at the discretion of the neuromodulation specialist. 14 of 16 patients were admitted to the epilepsy monitoring following implantation, during which time stimulation parameters were adjusted. Typically, low (2−10 Hz), moderate (40 Hz), and high (100−150 Hz) stimulation frequencies were tried. The location of cathodes and anodes were modified per available imaging with the assumption that cathodal stimulation is generally more effective than anodal stimulation. Stimulation settings were adjusted based on seizure frequency, the frequency of interical epileptiform discharges, and reported patient symptoms. CM stimulation parameter ranges at last follow up were 1.0–6.3 V for pulse amplitude, 60−150 μs for pulse width, and 2−100 Hz for pulse frequency. ANT stimulation parameter ranges were 2.1–6.3 V for pulse amplitude, 90−120 μs for pulse width, and 5−100 Hz for pulse frequency. When duty cycle function was active “on” period ranged from 0.1−15 sec while “off” period ranged from 0.1−50 sec. 15 of 16 patients were receiving bipolar stimulation at last recorded follow-up (Table 2), which is defined as each lead containing at least one anode and one cathode.

### 2.3. Variables and data collection

Baseline clinical characteristics were obtained from the electronic medical record (EMR). A standardized questionnaire was administered by phone to assess average monthly seizure frequency over the past three months, pre-DBS monthly seizure frequency, current annual convulsive seizure frequency and pre-DBS convulsive seizure frequency. In the questionnaire, convulsive seizures were defined as seizures in which patients shook their arms and legs rhythmically while unresponsive. For patients with very frequent baseline seizures we used an upper limit of 100 seizures per day given concern for reliable seizure counting. For patient perceived outcomes, the questionnaire included ten-point response scales to assess epilepsy severity, life satisfaction, and quality of sleep as used in other neuromodulation case series [17]. Verbal consent was provided. In case of underage patients or with documented intellectual/cognitive disability, parent(s) and/or legal guardian(s) completed the phone survey. One patient, who lives internationally, could not be reached for the questionnaire and seizure frequency was obtained from the EMR. Responders were defined as those with at least 50 % seizure frequency reduction. Follow up time represents the time elapsed from DBS implantation to the standardized questionnaire (or the last documented seizure frequency in the case of the single patient who was not available to complete the questionnaire). When assessing response to VNS prior to DBS implantation, subjective improvement was defined as the perception of improvement in seizure severity or frequency and not necessarily representing at least a 50 % reduction in seizure frequency. All questionnaire data were compared with available data from the EMR and were consistent.

### 2.4. Statistical analysis

All tests were performed in SPSS v.27 (IBM Corp., Armonk, NY, USA). Median and interquartile range (IQR) were reported for numerical variables and relative frequencies for categorical variables. Wilcoxon signed-rank and rank sum tests were performed for paired and non-paired comparisons, respectively. Fisher exact test was used for comparing categorical variables. P values < 0.05 were considered significant.

## 3. Results

Table 1 shows patients’ baseline characteristics. Median age at implantation was 18 y (IQR, 10−24 y). Follow up median time was 80 months (IQR, 37–97 mo). There were seven combined generalized and focal epilepsy patients (44 %), six generalized epilepsy patients (37 %), and three patients with focal epilepsy (19 %). Five patients had a diagnosis of Lennox-Gastaut syndrome (LGS) (31 %). Twelve patients (75 %) had VNS implanted before DBS, of which four (25 %) had documented subjective improvement. Two patients (12 %) continued active VNS stimulation following DBS initiation. Patients had previously tried a median of nine anti-seizure drugs (ASD) (IQR, 6–12) before DBS and were taking a median of three (IQR, 2–4) at the time of DBS implantation. Ketogenic diet had been used in 13 patients (81 %) with one (6%) patient on the diet at the time of DBS implantation.

**Fig. 2.**
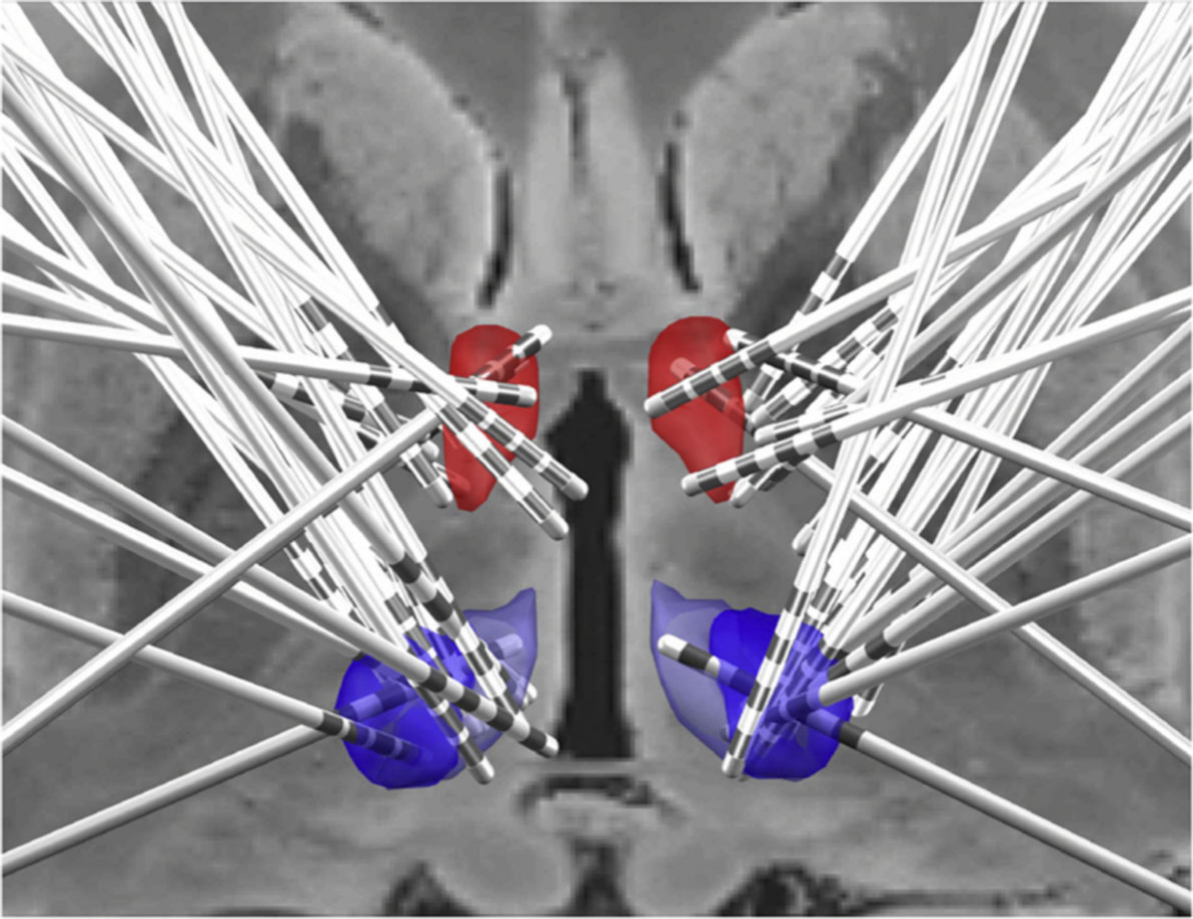
Reconstruction of electrode locations of 13/16 patients. ANT (7/10) – red, CM (13/16) – blue.

Regarding proximity of the leads to the intended target, 13 of 16 patients had imaging sufficient for lead localization. Shortest distance to target was calculated as the average of the shortest left and right electrode distance to either CM or ANT in mm. All patients with CM leads had at least one contact within 2.5 mm of the target bilaterally with a median shortest distance from target of 0.25 mm (IQR, 0.19–1.12 mm). For ANT leads, 80 % of patients had at least one contact within 2.5 mm of the target (70 % bilaterally, 10 % unilaterally), while 20 % did not have any ANT contacts within 2.5 mm of the target. No leads were repositioned. Median shortest distance from ANT target was 2.00 mm (IQR, 1.15–3.55 mm). The 2.5 mm is an arbitrary cutoff consistent with prior modeling data [18,19]. Shortest electrode distance from CM target was not significantly different in responders (9/13) vs. non responders (4/13), (0.25 mm; IQR, 0.18–1.17 vs. 0.28 mm; IQR, 0.21–0.88; p = 1.00) nor was shortest distance from ANT target different in responders (7/10) vs. non-responders (3/10), (1.31 mm; IQR, 0.99–3.48 vs. 2.59 mm; IQR, 2.56–3.17; p = 0.27). Table 2 contains information on individual electrode distance from target, and stimulation parameters at the last follow-up visit. Fig. 2 show a composite map of all implanted leads.

### 3.1. Seizure frequency

Baseline median seizure frequency was 323 sz/mo (IQR, 71–563 sz/mo). Patients reported a significant median seizure frequency reduction of 58 % (IQR, 13–87 %, p = 0.002). Patients did not report new seizure semiologies after DBS except for patient 12, who developed atonic seizures four months after implantation. Ten patients (63 %) were responders (≥50 % seizure frequency reduction). There was no difference in seizure frequency reduction or responder rate in the CM + ANT group compared to the CM only group (60 % vs. 56 %; p = 0.583 and 63 % vs. 60 %; p = 1.0, respectively). One patient reported increased seizure frequency by 15 %. For assessment of convulsive seizure frequency reduction, twelve patients (75 %) reported convulsive seizures at the time of DBS placement (see Table 1); of these patients, four reported at least a 50 % decrease in the frequency of convulsive seizures following DBS. However, this decrease was not a statistically significant reduction (6 sz/y; IQR, 0–90 vs.7 sz/y; IQR, 0–75; p = 0.173).

### 3.2. Subjective patient outcomes

Seizure severity improved in 62 % of patients (p = 0.034) (Fig. 1). Life satisfaction improved in 56 % of patients (p = 0.047). Quality of sleep did not improve significantly with 37 % of patients reporting a favorable change (p = 0.063); no patients reported more than a 1-point decline.

### 3.3. Adverse events

There were three DBS related adverse events (AE) (19 %). One patient had pulse generator rotation that required pocket revision. Another patient had complete system removal due to a pocket infection that extended to the leads. One patient had postprocedural transient left hemiparesis not associated with bleeding that resolved after one month. Stimulation was discontinued in two patients at the parents’ request due to a lack of perceived benefit.

## 4. Discussion

This study demonstrates that CM DBS with or without simultaneous ANT stimulation is feasible, safe, and associated with a significant seizure frequency reduction in this difficult-to-treat patient cohort with generalized, multifocal, posterior origin, and diffuse onset DRE. We observed a 58 % median seizure frequency reduction with 63 % responder rate with median follow up of 6.6 years. These results were not influenced by stimulation target. Two-thirds of our patients perceived less severe seizures and increased life satisfaction, which includes patients who had a <50 % seizure frequency reduction. In SANTE, quality of life improved by up to 48 % [8].

We focused on the management of a heterogeneous population using off-label FDA-approved devices in patients who are not normally enrolled in controlled neurostimulation trials. Although simultaneous neocortical plus CM RNS [20] and four-lead thalamic DBS have been previously reported [21], our group is the first to report simultaneous CM + ANT stimulation for the management of DRE, expanding on a prior case series of 4 patients [22]. Prospective controlled thalamic DBS studies demonstrate that ANT is an effective target for seizure control in focal epilepsy, especially for frontal and temporal onset seizures [8]. Evidence suggests CM works better for generalized epilepsy in well-defined patient profiles [10,11]. Multifocal onset seizures may benefit from stimulation at either target, although supporting evidence is weaker [7,23].

Whether simultaneous CM + ANT stimulation provides additional seizure control in severe DRE remains unknown. Previous work found that one of five patients with frontal epilepsy responded to CM stimulation [10]. In our cohort, two out of three patients with focal epilepsy were responders (patients 10 and 11). Possibly, adding ANT stimulation may desynchronize cortical and thalamocortical activity and reduce seizure frequency from zones involving frontal and temporal structures connected to the Papez circuit [24]. Nonetheless, there is evidence from a retrospective cohort that CM stimulation alone can provide up to 68 % seizure reduction for patients with multifocal onset seizures [23].

Our population includes pediatric and adult patients who have a severe phenotype with a median monthly seizure rate of 323, the majority of whom had multifocal and generalized onset seizures. Three patients had clearly identified atypical absence seizures and focal impaired awareness seizures. The coexistence of generalized onset and focal onset seizures is well-recongized in severe epilepsies and is recognized in the 2017 ILAE classification “Combined Generalized and Focal Epilepsies” [25]. The focal DRE patients had either posterior onset or diffuse temporal seizure onset. Our patients were markedly refractory to pharmacological therapy having tried a median of nine ASDs before DBS. VNS had been used in 75 % of patients and only a quarter of those had a favorable response. Due to their complexity and heterogeneity, these patients tend to be excluded from controlled trials. The SANTE trial excluded patients who had more than 10 seizures daily, which was the norm for our patient population [7]. Moreover, patients who had neither frontal lobe nor temporal lobe seizure onsets did not have a significant seizure reduction in SANTE due to a lack of statistical power [8]. This finding highlights the underrepresentation of patients with posterior onset and multifocal onset epilepsy in large RCTs.

To date, the largest CM-DBS controlled trial involved two centers with 11 patients; four patients with idiopathic generalized epilepsy and two with presumed symptomatic generalized epilepsy with normal head imaging had an average seizure frequency reduction of 77 % [10]. In contrast, 30 % of our study population had structural abnormalities and the majority had multifocal interictal discharges, which may account for the smaller seizure reduction that we observed.

Electrode location likely plays a role in DBS efficacy. In the case of CM stimulation for LGS, patients with >80 % seizure frequency reduction had electrodes within the anterolateral CM, whereas in those with <80 % reduction, electrodes tended more towards the superior, medial and posterior aspect of the CM [16]. For ANT stimulation, active contacts closer to the anterior half of the ANT were associated with improved seizure frequency reduction [26]. Here, we evaluated mean electrode distance from target and did not find a significant difference between responders and non-responders for either CM or ANT nuclei. Population heterogenity, small sample size, and the variability of stimulation parameters may account for the lack of statistical significance.

In terms of safety, 19 % of patients experienced AE that were attributable to DBS with 12.5 % requiring surgical intervention. The AE rate in our population is consistent with the SANTE trial data where 29.5 % of AE were considered to be DBS related [7]. One of our patients (6%) required system removal due to implant site infection. This is comparable to the 5 patients (4.5 %) that required complete system explanation after implant site infection in the long term follow up of the SANTE trial [8]. One patient developed a new semiology after DBS placement; however, this was deemed secondary to her generalized epilepsy phenotype and not as a side effect of DBS.

Limitations include small sample size and an uncontrolled and retrospective design that precludes drawing causal conclusions. For example, ASD medication changes as well as ongoing VNS therapy (*n* = 2) could certainly have contributed to positive outcomes. Despite this, for the 10 patients who experienced a significant seizure reduction, we attribute the response primarily to DBS. These patients experienced an initial response proximate to their implantation, and in our experience these kinds of reductions in seizure frequency are rare in these severe epilepsies. Patients were stimulated at the discretion of the specialist without a standardized protocol which may impact reproducibility. The evaluation of patient perceived outcomes with 10-point scales is limited and is not a substitute for more comprehensive epilepsy specific batteries such as the Liverpool Seizure Severity Scale (LSSS), or the Quality of Life in Epilepsy Inventories (QOLIE). The cross-sectional assessment of seizure frequency may introduce recall bias that we attempted to minimize by cross-validating with the EMR to avoid major discrepancies. Our assessment did not delineate seizure frequency for each seizure type as this was not deemed feasible retrospectively for these complex patients; post-treatment seizure frequency may represent a different composition of seizure types. Similarly, small numbers precluded subgroup analyses such as determining the benefit of adding ANT stimulation to CM stimulation for the treatment of generalized onset seizures. Finally, we note that 20 % of patients with ANT leads did not have any contacts within 2.5 mm of the target, which could contribute to reduced efficacy.

## 5. Conclusions

These results suggest deep brain stimulation of CM and CM + ANT significantly reduces seizure frequency in a majority of patients from a heterogenous cohort of children and adults with generalized, multifocal, posterior origin, and poorly localized epilepsy. A notable minority of patients did not show a response. Overall, patients perceived reduced epilepsy severity and improved life satisfaction. CM and CM + ANT implantations are safe, with comparable adverse event rates to prior studies of DBS for epilepsy. These are DRE patients with severe phenotypes who currently do not fit the populations where ANT or CM have been useful in controlled prospective trials. Our results should motivate future controlled studies to assess CM and CM + ANT DBS for generalized, combined generalized and focal, and poorly localized or posterior onset focal epilepsies.

## Figures and Tables

**Table 1:** Summary of Patient Characteristics and Results.

Patient / IPG	Sex / Age* (y)	Epilepsy onset	Epilepsy description	Seizures type	MRI findings	ASDs trialed pre-DBS/ASD at time of implant	F/U time (mo)	ASDs at last F/U	Total seizure frequency (sz/mo)		Convulsive seizure frequency (sz/y)		Comments
									re	post	re	post	
CM + ANT patients													
1 / Restore	F/6	1 mo	Generalized seizures, multifocal discharges	AT, GT, MYO	Bilateral parieto-occipital encephalomalacia	16 / (FBM)	86	FBM, VPA	450	525	NA	NA	DBS was turned off after 17 mo of stimulation; LGS
2 / Activa 2x	F/31	15 y	Generalized and focal seizures	AT, AA, GTCS	Non lesional	9 / (CLB, LEV, OXC)	50	CLB, OXC	3000	45	<1	<1	NA
3 / Activa 2x	M/49	6 mo	Generalized seizures, multifocal discharges	GT	Non lesional	6 / (CLB, Clzp, FMB, LEV, LTG, PHT, RUF)	39	CLB, Clzp, FMB, LEV, LTG, PHT, RUF	600	300	2	2	Callosotomy pre-DBS; Active VNS
4 / Restore	M/15	12 mo	Generalized and focal seizures	GT, FIAS, FAS	Non lesional	11 / (Clzp, LEV, LCM)	100	CLB LTG, VPA	60	10	NA	NA	IPG rotation requiring pocket revision
5 / Restore	F/19	10 mo	Generalized and focal seizures	FIAS, AA, GTCS	Non lesional	8 / (LEV, Clzp)	99	LEV, Clzp	3000	300	6	<1	NA
6 / Restore	F/6	11 mo	Generalized and focal seizures	FIAS, AA	Right hemispheric encephalomalacia	2 / (Clzp, FBM, OXC, VPA)	97	Clzp, FBM, OXC, VPA	105	60	0	12	R temporal lobectomy pre-DBS
7 / Restore	F/24	6 mo	Generalized and focal seizures	AA, FIAS, GTCS	Non lesional	6 / (CLB, LEV, RUF, VPA)	92	CLB, LEV, VPA	345	46	540	24	NA
8 / Activa 2x	M/10	5 years	Generalized and seizures focal	FIAS, AT, MYO, GTCS	Non lesional	7 / (CLB, LEV, PB, STP)	81	CLB, LEV, PB, STP	225	225	72	72	Callosotomy pre-DBS; SCN1A mutation; Lost to follow up
9 / Restore	F/16	9 y	Multifocal seizures	FBTCS	Left mesial temporal sclerosis	4 / (LEV, LCM, OXC)	97	FBM, LEV, OXC	8	6	96	76	L temporal lobectomy pre-DBS; DBS removed due to infection after 29 mo of stimulation; History of encephalitis
10 / Activa 2x	M/38	11 mo	Multifocal seizures	FIAS, FBGTCS	Non lesional	12 / (CLB, CZP, RUF)	78	CZP, RUF, VPA	10	1	52	2	NA
11 / Intellis	F/19	10 y	Generalized and focal seizures	AA, FIAS, FBGTCS	Left periventricular heterotopia	2 / (ACZ, CLB, LTG, TPM)	24	CLB, LTG, TPM	300	120	6	1	NA
CM only patients													
12 / Activa	F/22	2 mo	Generalized seizures	MYO, GT	Non lesional	12 / (CZP, FBM, VPA)	97	CLB, FBM	135	18	<1	<1	DBS turned off after 21 mo of stimulation; Developed AT after DBS
13 / Activa	M/10	3 y	Generalized seizures	GT, MYO, AA	Non lesional	8 / (CLB, FBM)	56	FBM, LTG	30	27	NA	NA	LGS
14 / Activa	F/10	4 mo	Generalized seizures	AT, GT, AA	Non lesional	14 / (PB)	36	PB, PRP	450	450	0	168	Active VNS; LGS
15 / Activa	M/14	1 mo	Generalized and focal seizures	FIAS, GTCS	Non lesional	11 / (CLB, FBM, LEV, PRP)	1	CLB, FBM, LEV, PRP	450	150	NA	NA	Callosotomy pre-DBS; Isodicentric chromosome 15
16 / Activa	M/20	3 mo	Generalized and focal seizures	AA, GTCS, FIAS, MYO	Bilateral pachygyria	9 / (CLB, FBM, LCM, PRP, VGB)	19	CBD, CLB, FBM, VGB	1350	600	45 daily	1080	ANT not placed due to financial constraints; LGS

AA, atypical absences; ACZ, acetazolamide; ANT, anterior thalamic nuclei; AT, atonic seizures; CLB, clobazam; Clzp, clorazepate; CM, centromedian thalamic nuclei; CZP, clonazepam; DBS, deep brain stimulation; FBM, felbamate; FBTCS, focal to bilateral tonic clonic seizures; FIAS, focal impaired awareness seizures; FAS focal preserved awareness seizures; F/U, follow up: GT, generalized tonic seizures; GTCS, generalized onset tonic clonic seizures; IPG, implantable pulse generator; LGS, Lennox Gastaut Syndrome; mo, months; LEV, levetiracetam; LCM, lacosamide; LTG, lamotrigine; MYO, myoclonic seizure; sz, seizure; OXC, oxcarbazepine; PB, phenobarbital; PHT, phenytoin; PRP, perampanel; RUF, rufinamide; Stim, stimulation; STP, stiripentol; TPM, topiramate; VGB, vigabatrin; VNS, vagus nerve stimulator; VPA; valproic acid.

* Age at DBS implantation.

**Table 2:** DBS lead configuration, distance from electrode to target by image reconstruction, and last recorded stimulator settings.

Patient / IPG	CM stimulation last recorded settings^·^; lead model	Right CM lead setting and distance from target (mm)		Left CM lead setting and distance from target (mm)		ANT stimulation last recorded settings^·^; lead model	Right ANT lead setting and distance from target (mm)		Left ANT lead setting and distance from target (mm)	
1 / Restore	4 V, 90 μs, 40 Hz; Medtronic 3387	12 +	0.2	8 +	0.9	4 V, 90 μs, 40 Hz; Medtronic 3387	4 −	6.2	0 −	1.3
		13 −	0.2	9 −	0.3		5 −	7.8	1 −	2.9
		14 −	1.1	10 −	1.9		6 −	9.5	2 −	5.8
		15 +	4.0	11 +	5.3		7 +	11.8	3 +	9.1
2 / Activa 2x	2 V, 90 μs, 100 Hz; Medtronic 3389	12 −	0.2	8 −	0.3	4 V, 90 μs, 40 Hz; Medtronic 3389	4 −	8.0	0 −	9.5
		13 −	0.3	9 −	0.2		5 −	9.3	1 −	11.4
		14 −	0.3	10 −	0.3		6 −	10.9	2 −	13.3
		15 +	1.7	11 +	0.9		7 +	12.6	3 +	15.2
3 / Activa 2x	3 V, 210 μs, 7 Hz; Medtronic 3387	12 −	3.3	8 −	3.3	3 V, 210 μs, 7 Hz; Medtronic 3389	4 −	1.7	0 −	0.4
		13 −	0.2	9 −	0.4		5 −	3.0	1 −	0.3
		14 −	0.2	10 −	0.1		6 −	4.4	2 −	0.4
		15 +	0.5	11 +	0.2		7 +	5.5	3 +	0.3
4 / Restore	6.9 V, 90 μs, 8 Hz; Medtronic 3387	12 −	0.2	8 −	2.5	6.9 V, 90 μs, 8 Hz; Medtronic 3387	4 −	0.3	0 −	2.6
		13 −	0.0	9 −	2.3		5 −	1.1	1 −	4.9
		14 +	0.7	10 +	3.2		6 +	3.5	2 +	7.6
		15 o	3.3	11 o	4.9		7 o	6.0	3 o	10.5
5 / Restore	6 V, 120 μs, 15 Hz; Medtronic 3387	12 o	1.4	8	2.0	6 V, 120 μs, 15 Hz; Medtronic 3387	4 o	1.9	0 o	3.0
		13 −	2.1	9	0.2		5 o	3.6	1 o	1.0
		14 +	4.1	10	1.0		6 −	5.9	2 −	0.8
		15 o	6.8	11	3.8		7 +	8.9	3 +	3.1
6 / Restore	5 V, 90 μs, 7 Hz; Medtronic 3387	12 +	NA	8 +	NA	5 V, 90 μs, 7 Hz; Medtronic 3387	4 −	NA	0 +	NA
		13 −		9 −			5 −		1 −	
		14 +		10 +			6 +		2 +	
		15 o		11 o			7 o		3 o	
7 / Restore	2.2 V, 90 μs, 40 Hz; Medtronic 3387	12 −	0.2	8 −	0.2	2.2 V, 90 μs, 40 Hz; Medtronic 3387	4 −	2.0	0 −	0.4
		13 −	1.8	9 −	0.5		5 −	3.6	1 −	0.8
		14 −	4.4	10 −	3.3		6 +	5.5	2 +	3.0
		15 +	7.5	11 +	6.3		7 o	7.8	3 o	5.4
8 / Activa 2x	4 V, 90 μs, 100 Hz; cycling 10 sec on, 50 sec off; Medtronic 3387	12 o	0.3	8 o	1.8	4 V, 90 μs, 100 Hz; Medtronic 3387	4o	3.0	0 o	2.2
		13 o	0.9	9 o	2.3		5 −	5.8	1 −	2.4
		14 −	3.1	10 −	3.6		6 +	8.9	2 +	3.9
		15 +	6.1	11 +	6.1		7 o	12.1	3 o	6.3
9 / Restore	4 V, 120 μs, 100 Hz; cycling 15 sec on, 45 sec off; Medtronic 3387	12 o	0.3	8 o	0.3	4 V, 120 μs, 100 Hz; cycling 15 sec ON, 45 sec off; Medtronic 3387	4 o	2.7	0 o	2.4
		13 −	0.3	9 −	1.3		5 −	4.7	1 −	4.0
		14 −	1.5	10 −	4.0		6 −	7.1	2 −	6.1
		15 +	4.4	11 +	6.8		7 +	9.6	3 +	8.4
10 / Activa 2x	1V, 60 μs, 100 Hz; Medtronic 3387	12 −	3.1	8 −	0.3	4 V, 120 μs, 5 Hz; Medtronic 3389	4 −	3.7	0 −	3.4
		13 −	2.7	9 −	0.6		5 −	4.3	1 −	3.3
		14 +	3.8	10 +	2.8		6 +	4.7	2 +	4.1
		15o	5.8	11 o	5.6		7 o	5.5	3 o	5.0
11 / Intellis	2.5 mA / lead, 120 μs, 2 Hz; Medtronic 3387	12 +	0.8	8 +	1.0	2.5 mA / lead, 120 μs, 7 Hz; Medtronic 3389	4 +	0.2	0 +	0.4
		13 −	0.3	9 −	0.2		5 −	0.3	1 −	0.3
		14 −	0.1	10 −	0.3		6 −	1.6	2 −	0.9
		15 +	1.1	11 +	0.2		7 +	2.8	3 +	2.0
12 / Activa	4 V, 120 |μS, 100 Hz; Medtronic 3387	8 − C +	NA	0 − C +	NA	-	-		-	
		9 − C +		1 − C +						
		10 o		2 o						
		11 o		3 o						
13 / Activa	2.5 V, 90 μs, 100 Hz; Medtronic 3389	8 −	2.7	0 −	0.3	-	-		-	
		9 −	0.5	1 −	0.3					
		10 −	0.2	2 −	0.2					
		11 +	0.1	3 +	2.2					
14 / Activa	5 V, 210 μs, 3 Hz; Medtronic 3389	8 +	NA	0 +	NA	-	-		-	
		9 +		1 +						
		10 −		2 −						
		11 −		3 −						
15 / Activa	4.5 V, 90 μs, 100 Hz; Medtronic 3389	8 −	0.5	0 −	0.9	-	-		-	
		9 −	0.2	1 −	0.3					
		10 −	0.3	2 −	0.6					
		11 +	0.2	3 +	0.7					
16 / Activa	3.5 V, 90 μs, 40 Hz; Medtronic 3387	8 +	0.2	0 +	4.4	-	-		-	
		9 −	0.2	1 −	2.2					
		10 −	1.2	2 −	2.6					
		11 +	4.7	3 +	3.4					

ANT, anterior thalamic nucleus; CM, centromedian thalamic nucleus; Hz, hertz; IPG, implantable pulse generator; NA, not available; mA, milliamperes; μs, micro-seconds.

+, positive electrode, −, negative electrode; o, inactive electrode; C +, IPG as positive electrode.

Electrodes 0, 4, 8 and 12 are tip of the 4-electrode lead.

· Stimulation is continuous if no cycling parameters are specified.
